# Lack of agreement between measured and self-reported distance from public green parks in Glasgow, Scotland

**DOI:** 10.1186/1479-5868-5-26

**Published:** 2008-05-04

**Authors:** Sally Macintyre, Laura Macdonald, Anne Ellaway

**Affiliations:** 1 MRC Social & Public Health Sciences Unit, 4 Lilybank Gardens, Glasgow, G12 8RZ, UK

## Abstract

**Background:**

Reviews have reported mixed findings for associations between physical activity and proximity to a range of environmental resources. Initially most studies used self reported proximity, but more are now using GIS techniques to measure proximity objectively. We know little about the extent of agreement between self reported and directly measured proximity of the same resource.

**Methods:**

We used previously collected data in a community survey in Glasgow in which 658 respondents aged around 40 and 60 were asked whether they lived within half a mile of a public park. We compared their answers with GIS measures of whether there was a park within a half mile service area of their home (and whether their home was within a half mile crow fly buffer of a park).

**Results:**

Agreement was poor; percentage agreement between measured network distance and reported residence within 0.5 miles of a park was 62.0%, and the kappa value was 0.095. Agreement was no higher than poor in any socio-demographic subgroup, or when using crow fly buffers instead of service areas.

**Conclusion:**

One should be cautious about assuming that respondents' self reports of proximity to a resource are a valid proxy for actual distance, or vice versa. Further research is needed to establish whether actual or self-reported proximity predict physical activity or other behaviours, and if so which is the strongest predictor. Further, qualitative study, also needs to examine the basis of people's judgements about the location of resources, and the possibility that these are shaped by their social and personal significance.

## Background

In recent years there has been considerable interest in the potential health benefits of public parks, other green spaces, or greenery in general. Much of this work has focused on possible associations between proximity to parks and physical activity [1-6], but some has considered broader, public health and mental health aspects of proximity to parks [7-9]. Initially most of the investigations into associations between health or physical activity and proximity to parks was based on respondents' self-reported accounts of their access to parks [10,11], but more recently Geographic Information Systems (GIS) (computerised systems which manage spatially referenced data [12]) or trained observers have been used to measure access to parks or the greenness of the immediate environment objectively [4-6,13]. Reviews have found variability between studies in observed associations between physical activity and proximity to parks and other potential activity settings [11,14-16].

One reason for this variability may be the use of different measures of proximity to settings in different studies, and in particular to the mixture of self-reported and GIS or observer-assessed distance. Sallis et al, for example, found that objective measures of access to exercise facilities were associated with physical activity while respondents' reports of access were not, and there was no significant correlation between the directly measured and the self reported [17]. Tilt et al found that self-reported measured greenness in the neighbourhood was significantly associated with walking trips but objective greenness was not; objective accessibility of a number of resources was associated with walking trips but the authors do not report whether or not self-reported accessibility was. Objective accessibility of 17 destinations (banks, coffee shops, parks etc) within 0.4 miles (643.7 metres) was correlated with self-reported accessibility within 0.5 miles (804.7 metres) (R = 0.314, p < 0.0001). Controlling for socio-demographic factors, the regression of self-reported accessibility on objective accessibility produced R^2 ^of 0.110 (i.e. 11% explained variance). Eighty-one percent of respondents to a community survey said they lived within 0.5 miles of a park, whereas 62% were measured by GIS as living within 0.4 miles of a park; kappa value for agreement between objective and self-reported accessibility to a park was 0.154 [18].

Kirtland et al compared directly measured and respondent assessed proximity to a range of environmental features in South Carolina, using two spatial scales (neighbourhood, within a 0.5 mile radius of the respondent's home, and community, within a ten mile radius). Levels of agreement between objective and self-reported access were fair to low for neighbourhood items (kappa = -0.02 to 0.37, with higher values, [0.19 to 0.37], for access to sidewalks, public recreation facilities, and streetlights), and low to slight for community items (kappa -0.07 to 0.25, with 0.25 being for the presence or absence of shopping malls). Kappa was 0.01 for whether there were any parks, playgrounds or sports fields in the community. Levels of agreement varied by physical activity levels; for example agreement on the presence of recreational facilities was higher among active respondents than insufficiently active or inactive respondents. The authors suggested that the relatively low level of agreement might relate to people's inability to judge distances, and their personal behaviours (for example transportation routes), beliefs and values [19].

In a study of two neighbourhoods in Adelaide, South Australia McCormack et al presented participants with a list of nine destinations including a park and asked them to report how long it would take to get from their home to the facility if they walked. Self-reported distance (based on an average walking speed of 4.5 km (2.8 miles)/hour) was calculated based on these self-reported walking times and then compared with the actual distance (using road network analysis). There was relatively poor agreement between the objective and self-reported measures of distance [20].

Sallis et al's study used self-reported convenience of 15 exercise facilities, and noted little correlation between that and measured density of all (aerobic) exercise facilities within 1 km of each resident's home. This is perhaps not surprising since self-reported *convenience *and local *density *may not be measuring the same thing [17]. Tilt et al's study used different distance measures for objective (0.4 mile) and self-reported (0.5 mile) access [18]. The Kirtland study used rather general measures, some of them qualitative rather than binary (e.g. standard of maintenance of sidewalk, safety, heaviness of traffic, quality of street lighting, whether unattended dogs were a problem), and the question on parks related to a ten mile radius rather than 0.5 mile radius [19]. McCormack et al noted that a limitation of their study was that assumptions were made about the average walking speed of their study participants and this may have affected their results [20]. Thus these studies were not examining self-reported and objectively measured versions of exactly the same variables.

In this paper we attempt to fill this gap by re-analysing an existing data set to address two questions: what is the extent of agreement between self-reported and objective measures of the same variable (whether or not the respondent lived within 0.5 miles of a public green open space)?; and does agreement between the self reported and directly assessed measures vary by socio-demographic and behavioural variables (given that previous literature suggests variation in perceptions of the local environment by such characteristics [21-25])?

## Methods

We used data from the locality component of the 'The West of Scotland Twenty-07 Study: Health in the Community', the aim of which is to explore the social processes which produce or maintain differences in health by key social positions, in particular, by gender, age, social class, ethnicity, family composition and area of residence. The study is following up three cohorts (born in 1932, 1952 and 1972) using wide ranging home-based interviews and postal questionnaires. Respondents were aged 15, 35 and 55 years at first contact in 1987/88 [26].

**Figure 1:**
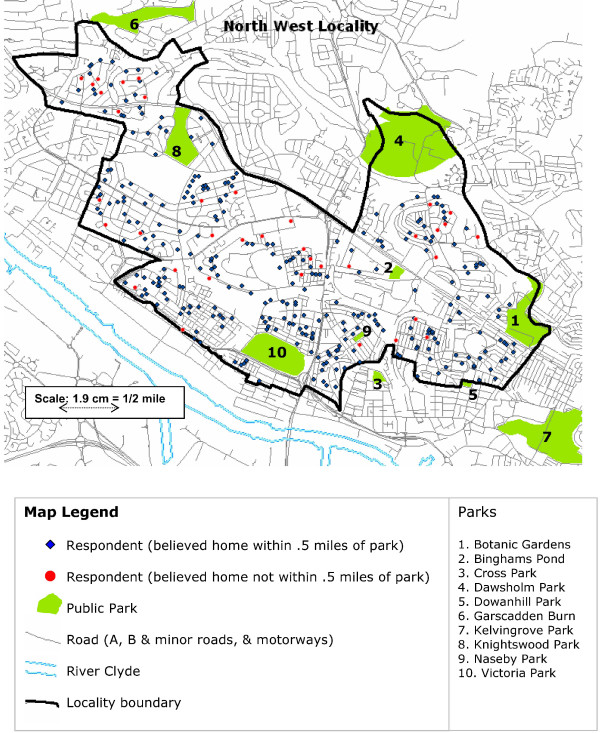
**Map of North West locality, Glasgow**. Location of public parks and of home addresses of survey respondents.

**Figure 2:**
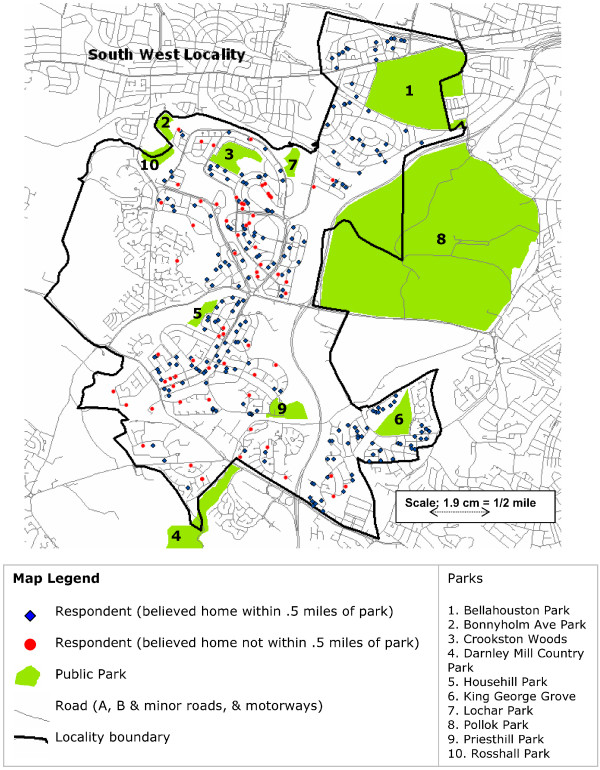
**Map of South West locality, Glasgow**. Location of public parks and of home addresses of survey respondents.

The locality component involves relatively intensive study of two areas of Glasgow City with contrasting socio-residential characteristics – the North West (NW) and South West (SW). (See Figures 1 and 2). The NW is relatively advantaged (as measured by census level indicators such as unemployment rates, housing tenure, occupation, and car ownership) and has better health indices, while the SW is relatively disadvantaged with worse health indices. Neither locality is at the extreme of health or social advantage in Glasgow [27,28]. Data from the respondents in the locality sample have been gathered on four occasions so far, with interviews in 1987, 1992, 2000–3, and a postal survey in 1997. This analysis uses data from the 1992 interviews which involved the two oldest cohorts, then aged 40 and 60. At interview respondents were asked a range of structured questions about how they perceived their local area, including a question on whether they lived 'within walking distance (half a mile) of a park'. We do not have data on the test retest reliability of this question, and we are not aware of any reliability data on this specific question in the literature. Test retest reliability of general perceptions of the environment of relevance to physical activity has been shown in other studies to vary by the item in question, and to be moderate to high [29-31].

At the 1992 sweep 690 respondents lived within the locality boundaries. Thirty two of them were excluded from this analysis; four because they did not answer the question on whether they lived within half a mile of a park, and 28 because their addresses in 1992 (16 from the NW and 12 from the SW) were not recognized when matched to the Ordnance Survey address point data because of inaccurate or out-of-date address data. This analysis therefore included 658 respondents; 352 within the NW locality and 306 in the SW.

Ordnance Survey maps (including addresses and roads, tracks, paths, buildings etc), were obtained, and ESRI ArcMap 9.1 was used to geocode the location of every respondent by their unit postcode (the smallest level of postal geography in the UK) and their point address (i.e. street number and name). All the twenty public green parks owned by Glasgow City Council within the boundaries of the localities, and half a mile outwith the boundaries, were also mapped. (Note that we did not include publicly maintained hard based spaces such as skate board or BMX parks since in the UK these would not normally be what people think of by the term 'parks'). According to the City Council, these comprised four City parks, four District parks, eleven Local parks and one wooded park; see Figures 1 and 2. Most of these were of nineteenth century origin and the one most recently taken over by the City was Pollok Park, which was gifted to the City for public use in 1966. Thus all the parks predated the question asked of respondents in 1992, and the geocoding of their homes, by at least 26 years. The ArcMap software was used to create service areas, with a network (i.e. road or path) distance of 1/2 mile, around each respondent's home. The presence or absence of a park within the 1/2 mile service area around each respondent home was determined (i.e. any part of the boundary of the park would need to intersect with the service area of the respondent to be regarded as present). We also measured whether each respondent lived within a half mile crow fly buffer (i.e. straight line Euclidean distance) from the edge of each park.

SPSS 14.0 for Windows was used to investigate the agreement between objective and self-reported proximity to public parks. Kappa values, and percentage agreement and disagreement, were calculated. We used the conventional cut offs for kappa of < 0.20 = poor, 0.21–0.40 = fair, 0.41–0.60 = moderate, 0.61–0.80 = good, and 0.81–1.00 = very good [32]. Analysis was undertaken for all respondents together, and also by locality; sex; age; head of household occupational social class (using the UK Registrar General's Classification of Occupations [33] divided here into non-manual [professional and white collar], III manual [skilled blue collar] and IV/V manual [semi and unskilled blue collar]); deprivation of local small area (divided into quintiles using the Scottish Index of Multiple Deprivation [34]); whether they reported having taken a walk of more than 2 miles in the previous year (and whether that was in or outside their local area); and having a car or dog in the household. We have no data on the reliability or validity of the responses to the question on walking two miles in the last year. We were unable to control for or stratify by length of residence in the area, since although we had information on how long respondents had lived at their address when interviewed in 1992 (40 year old cohort mean 10.75, years, 60 year old cohort mean 18.5 years), we did not have information on their previous address so cannot distinguish between those who moved within and between areas.

## Results

Using the GIS service area measure, 61.7% respondents (56.3% of the residents in the NW, and 68.0% in the SW), lived 0.5 mile or less from a public park. The majority of respondents (84.2% overall; 89.5% in the NW and 78.1% in the SW) reported living within half a mile of a park. Overall, the agreement between measured and reported residence within 0.5 miles of park was 62.0%, and the kappa value was 0.095, which is considered poor [32] (see Table 1).

**Table 1:** Self reported living within half a mile of a park, by measured distance to a park.

**All respondents**	**Lives within half a mile of a park**		**Lives more than half a mile from a park**		**Total**	
	**%**	**N**	**%**	**N**	**%**	**N**

**Believe they live within half a mile of a park**	87.4 (64.1*)	355	79.0 (35.9*)	199	84.2 (100.0*)	554
**Believe they do not live within half a mile of a park**	12.6 (49.0*)	51	21.0 (51.0*)	53	15.8 (100.0*)	104
**Total**	100.0 (61.7*)	406	100.0 (38.3*)	252	100.0 (100.0*)	658

* Row percentage (i.e. of respondents' perceptions)

Kappa Value = 0.095, Approx Sig = 0.004

Agreement 62.0% disagreement 38.0%

There was little difference between males and females, with agreement slightly higher for males than for females. Agreement was higher for the 40 year olds than for the 60 year olds, and in the SW compared to the NW, but not substantially so. For occupational social class, there was highest agreement within the non-manual (white collar) group, and similarly, agreement was highest in the least deprived quintile of area deprivation. (see Table 2).

**Table 2:** Agreement between self reported and directly measured distance of half a mile to a park, by sex, cohort, locality, class, area deprivation, car ownership, dog ownership, walking 2+ miles in previous year, and walking 2+ miles in previous year in area.

		**N**	**Kappa Value**	**% agreement**
**Sex**	**Males**	295	0.109	63.1
	**Females**	363	0.084	61.2
**Cohort**	**40 year olds**	303	0.164	65.7
	**60 year olds**	355	0.038	58.9
**Locality**	**North West**	352	0.111	59.9
	**South West**	306	0.107	64.4
**Social Class**	**I/II/III non-manual**	309	0.125	65.0
	**III manual**	143	0.089	59.4
	**IV/V manual**	140	0.040	60.0
**Area Deprivation**	**Quintile 1**	133	0.108	72.1
	**Quintile 2**	133	0.112	57.9
	**Quintile 3**	132	0.100	50.8
	**Quintile 4**	130	-0.047	62.3
	**Quintile 5**	130	0.136	66.9
**Car ownership**	**Owner**	394	0.079	60.7
	**Non-owner**	264	0.133	64.0
**Dog ownership**	**Owner**	160	0.166	64.4
	**Non-owner**	496	0.086	61.3
**Walked 2+ miles (previous year)**	**Walker**	428	0.060	61.9
	**Non-walker**	228	0.144	62.3
**Walked 2+ miles in area**	**Walker in area**	315	0.044	62.2
**(previous year)**	**Walker outside area**	112	0.107	61.6

Agreement was slightly higher for households who did not own a car or have a dog (both groups who might be expected to walk about their local areas more) than for those who did not. There was however little difference between respondents who reported having taken a walk for more than two miles in the previous year and those who did not, nor was there much difference whether their walks were in or outside their local area (see Table 2). In none of the socio-demographic, asset ownership or behavioural subgroups was agreement any better than 'poor'.

## Discussion and Conclusion

There was relatively poor agreement between our GIS measure and respondents' perceptions of living near a public park. Nearly thirteen per cent of respondents whose homes we measured as being within half a mile of a park said they did not live near to a park; conversely, 79% of those we measured as living further than half a mile from a park said they did live within half a mile of a park.

Thus one should be cautious about assuming equivalence between such measures, i.e. that respondents' perceptions are a valid proxy for actual distance or vice versa. While lack of correlation between GIS or observer measured and respondent self-reported access to facilities and resources has been noted before, previous studies [17-20] have not compared observed and self-reported access to the same, single, resource using the same distance measures, and it might therefore have been thought that there would be more congruence when directly comparing like with like.

There were no noteworthy or consistent differences in agreement by car or dog ownership, or by walking, which we had hypothesised might be related to agreement (car owners being less, and dog owners and walkers more, accurate in estimates of access to local parks), nor by socio-demographic characteristics. Unfortunately we were not able to examine whether there were any differences by length of residence.

It might be objected that when thinking about proximity to a park respondents are not thinking of precise, road based network distances, but of more general proximity; however, our crow fly measures showed similar lack of agreement (63.4%, kappa 0.096). We only asked about a half mile distance so were unable to compare the agreement on this measure with that which might be observed with a different distance e.g. a mile. It is striking that several respondents, particularly in the SW (Figure 2) lived very close to a park but did not report doing so. Because our data were collected from a structured survey instrument it is not possible for us to analyse what respondents thought of as "a park", and the extent to which this corresponds with what local government and official maps define as parks in the UK.

It may be that what is important for people's perceptions is not actual or potential proximity but some sort of symbolic proximity; for example because a park is not somewhere they ever go, or which they feel is suitable for people like them, it may not be in a person's perceptual field. A recent qualitative study of the opening of a new supermarket in a deprived area of Glasgow found that some local residents did not see the new supermarket as being 'for them' even though it was very close and sold affordable food at low prices [35]; it is possible that similar processes may be at work in relation to parks. For example, two of the parks which had residents living in close proximity who said they were not within half a mile of a park, were relatively 'wild' rather than 'manicured' (Crookston Woods and Darnley Mill Country Park, see Figure 2); it is possible that what physical activity advocates or town planners think of as attractive, public, accessible, natural environments are not seen as such by all local residents, who may instead think of them as alien or frightening [36], or simply not anywhere they would go.

Thus researchers interested in associations between proximity to resources and use of them, or other behaviours (for example, in this case physical activity), should not assume that self-reported proximity is the same as actual proximity. Further work needs to be done to establish whether or not actual or self-reported proximity predict physical activity or other behaviours, and if so which is the strongest predictor. Some authors have noted that the match between actual and self-reported distance may be influenced by the perceived quality (e.g. attractiveness) of the end point of the destination [37,38]. Further, qualitative study, therefore needs to examine the basis of people's judgements about the location of resources and the possibility that these are shaped by their social and personal significance.

## Competing interests

The authors declare that they have no competing interests.

## Authors' contributions

SM thought of the research question and presented an earlier version of this analysis at the 2004 ISBNPA meeting in Washington, and wrote successive drafts of the paper. LM did the mapping and data analysis and commented on all drafts. AE contributed to the conception and discussion of the analysis and to all drafts. SM is guarantor. All authors have read and approved the final manuscript.
